# Perception of the Potential for Interaction in Social Scenes

**DOI:** 10.1177/20416695211040237

**Published:** 2021-09-23

**Authors:** Roy S. Hessels, Jeroen S. Benjamins, Andrea J. van Doorn, Jan J. Koenderink, Ignace T. C. Hooge

**Affiliations:** Experimental Psychology, Helmholtz Institute 8125 , Utrecht University, Utrecht, the Netherlands; Experimental Psychology, Helmholtz Institute, 8125 Utrecht University, Utrecht, the Netherlands; Social, Health and Organisational Psychology, Utrecht University, Utrecht, the Netherlands; Experimental Psychology, Helmholtz Institute, 8125 Utrecht University, Utrecht, the Netherlands

**Keywords:** interaction, social scenes, gist perception, ensemble perception, presence

## Abstract

In urban environments, humans often encounter other people that may engage one in interaction. How do humans perceive such invitations to interact at a glance? We briefly presented participants with pictures of actors carrying out one of 11 behaviors (e.g., waving or looking at a phone) at four camera-actor distances. Participants were asked to describe what they might do in such a situation, how they decided, and what stood out most in the photograph. In addition, participants rated how likely they deemed interaction to take place. Participants formulated clear responses about how they might act. We show convincingly that what participants would do depended on the depicted behavior, but not the camera-actor distance. The likeliness to interact ratings depended both on the depicted behavior and the camera-actor distance. We conclude that humans perceive the “gist” of photographs and that various aspects of the actor, action, and context depicted in photographs are subjectively available at a glance. Our conclusions are discussed in the context of scene perception, social robotics, and intercultural differences.

In urban environments, social encounters are commonplace. When navigating crowds or walking into office buildings, supermarkets, and so forth, one inevitably encounters other human beings that may engage one in interaction. Others may want to exchange a greeting, shake hands, or engage in a brief chat. Conversely, others might ignore one altogether. How and when do humans perceive (or apperceive) such invitations or intentions to interact? One expects that this occurs at a glance (cf. Fei-Fei et al., 2007). Although this question has not been addressed directly, substantial research has been conducted on what humans can infer about others.

In general, humans categorize others (i.e., estimate what “kind” of person another is, cf. Cantor & Mischel, 1979) and can estimate aspects of their personality (Kenny et al., 1994; Zebrowitz & Collins, 1997), sexual availability (Gangestad et al., 1992), or what they think or feel (Ickes et al., 1990) from photographs or brief videos (Berry & Misovich, 1994). Research on the so-called *accuracy of social perception* has primarily focused on aspects that are relevant for long-term human interaction (e.g., personality traits) or survival (e.g., sexual availability, social dominance, see Zebrowitz & Collins, 1997, for a good starting point on this topic). Yet, this does not reveal how and when invitations or intentions to interact are perceived, that is, perception for human interaction in the short term. One potential exception is research on the perception of another’s intentions in sports, for example in cricket (Müller et al., 2006, 2010), tennis (Farrow & Abernethy, 2003), or soccer (Diaz et al., 2012).

From an inferential perspective (e.g., Brunswik, 1955), one may ask what cues are informative for the perception of the invitation or intention to interact. A prime example is the gaze direction of another person, on which a lot of work has been conducted (e.g., Anstis, 2018; Gibson & Pick, 1963; Todorović, 2006; von Cranach & Ellgring, 1973). Sweeny and Whitney (2017) aptly illustrate the relevance of perceiving another’s gaze direction for potential interaction:

Perceiving a person’s gaze direction is *critical for understanding and predicting their behaviors and intentions*… Perceiving when a person is looking directly at you is particularly important because it is a strong predictor that a social interaction may occur (Emery, 2000). (p. 67) [emphasis ours]

Emery (2000) has shown that various vertebrate species (including reptiles, birds, and mammals) perceive and use the direction of conspecifics’ gaze in establishing or regulating social interactions. Other cues that may be deemed relevant are, for example, facial expression, body posture, heading direction, or body kinematics.

In what is collectively known as ensemble perception (Whitney & Yamanashi Leib, 2018), it has been shown that humans perceive the average identity (Yamanashi Leib et al., 2014), gaze direction (Sweeny & Whitney, 2014), facial expression (Haberman & Whitney, 2007, 2009), or heading direction (Sweeny et al., 2013) in brief displays containing many elements (e.g., up to 16 faces or 12 point-light displays). Interestingly, observers perceive the average facial expression, but not the facial expression of any of the individual faces for briefly (50 ms) presented displays (Li et al., 2016). Similarly, humans can perceive the “gist”^1^ of rapidly presented photographs of natural scenes (e.g., Oliva, 2005; Thorpe et al., 1996). Of relevance to the perception of invitations or intentions to interact, Vanmarcke and Wagemans (2015) have shown that observers can accurately judge whether a photograph displays a positive or negative interaction (e.g., two friends sitting together) after a brief (83 ms) presentation. Scene gist also has contextual effects on, for example, the perception of facial and bodily expressions (Kret & de Gelder, 2010; Righart & De Gelder, 2008).

Regarding body kinematics, Blake and Shiffrar (2007) reviewed research showing that humans can perceive, for example, identity, personality traits, social dominance, but also vulnerability to attack from point-light displays of human motion (pp. 57–58). These point-light displays isolate body kinematics from, for example, facial expression or contextual information (Johansson, 1973). Using these displays, Dittrich (1993) showed that observers can recognize certain social actions (dancing, boxing, greeting, or threatening) above chance. Similarly, Dittrich et al. (1996) showed that observers can recognize emotion portrayed by expert dancers. However, presentation or recognition times are around 4 to 5 seconds, which can hardly be considered “at a glance.”

The studies here described make it clear that humans can perceive many cues that are potentially relevant for whether another person invites one or intends to interact, even under brief or degraded presentation. Yet, they only bear on the question of how and when humans perceive invitations or intentions to interact in an indirect manner. For one, a social encounter may be Gestalt-like, different from the sum of its parts. One wonders whether situations are conceivable where a combination of certain cues that individually may suggest an upcoming interaction together suggest that interaction is not likely to occur. Second, the *accuracy of social perception* suggests a fact of the matter, which does not exist for potential upcoming social encounters. Once one knows for sure whether the other invites one or intends to interact, one has either acted upon it or the moment has passed.^2^ Thus, an experimental phenomenological approach may be needed (see e.g., Albertazzi, 2013; Koenderink, 2014, 2019): When do human observers perceive that another invites one or intends to interact? When do observers agree (consensus) or not? How is this related to the distance to the other person, the context, but also, for example, distortions in time (cf. Koenderink et al., 2020)? To be clear, it is not about communication, it is about apperception of the situation and how this might guide action.

Such an experimental phenomenological approach to the perception of invitations or intentions to interact is first and foremost relevant to the study of (social) vision. Yet, it may also have practical implications in social robotics. Peters et al. (2005), for example, are interested in interaction between humans and embodied conversational agents. They write: “We model engagement opening as something that may start at a distance and may not initially involve an explicit commitment to engage, such as the use of a greeting utterance” (p. 233). Thus, there is clearly an interest in the perception of the invitation or intention to interact, and how this may unfold over distance to the observer, in social robotics. Peters (2005) presented a model that aimed to estimate human interest in interaction based on the direction of attention or “directed gestures” (facial expression or speech) (see also Gao et al., 2019, for relevant work on this topic). Although no evaluation results were reported, Peters (2005) aimed to evaluate his model by presenting users with a view of a mobile agent and then asking questions such as “how interested are they in you?,” and “do they want to interact?” Our work can provide insights into the circumstances under which humans perceive invitations or intentions to interact, beyond single cues such as gaze direction.

In the current study, we investigated what humans perceive in terms of the potential for interaction in social scenes under brief presentation durations (500 ms, or “at a glance”). We investigated for what scenes interaction was deemed likely, and how one might respond in such a situation. We used photographs that depict prototypical invitations and noninvitations for the context of most of our participants (the Netherlands; details follow below), which we expected to yield large differences in the perceived likeliness to interact. However, we did not assume a fact of the matter (correct/incorrect), but investigated under what circumstances participants expected interaction to occur or not. The behaviors were chosen partly on our previous work on looking behavior during potential interactions (Hessels et al.,2020). We chose a set that included some behaviors that may intuitively invite interaction and some that intuitively do not invite interaction at all. We excluded behaviors containing technical (e.g., traffic control hand signals) or coded gestures (e.g., sign language) (cf. Morris, 2002). Each behavior was photographed at one of four distances, to investigate the relation between camera-actor distance and the perception of invitations to interact. The distances were chosen such that they resulted in a close-up, upper body shot, full body shot, and long shot. We used both qualitative and quantitative techniques to answer our research question.

## Method

### Participants

Participants were recruited among colleagues and students through the authors’ network; 52 participants (34 female, 18 male) completed the experiment. Median age was 26 years (range 19–56 years). All participants reported normal or corrected-to-normal vision, with the exception of one participant who reported needing correction of –0.5 to –1.0 diopter. This research project does not belong to the regimen of the Dutch Act on Medical Research Involving Human Subjects, and therefore, there is no need for approval of a Medical Ethics Committee. Nonetheless, the study was conducted in accordance with the Ethical Principles for Medical Research Involving Human Subjects (World Medical Association Declaration of Helsinki) where applicable and approved by the Ethics Committee of the Faculty of Social and Behavioral Sciences at Utrecht University (protocol 20-522). All participants gave informed consent prior to starting the experiment.

### Stimuli

In this experiment, we used pictures of 11 actors (5 female, 6 male, age range approximately 25–78 years). Among the 11 actors were 4 of the authors. Each actor portrayed the following 11 behaviors (the shorthand descriptions in parentheses are used throughout the paper):

1. Looking at their phone (Phone)
2. Looking straight ahead (Look ahead)
3. Standing still and looking away (Turned away)
4. Trying to maintain distance (Hold off)
5. Waving (Wave)
6. Giving a fist bump (Fist bump)
7. Showing a thumbs up (Thumbs up)
8. Mimicking lighting a cigarette (Ask for lighter)
9. Showing a flyer (Flyer)
10. Signing someone to follow him/her (Follow)
11. Cheering with fists raised (Cheering)

**Figure 1.**
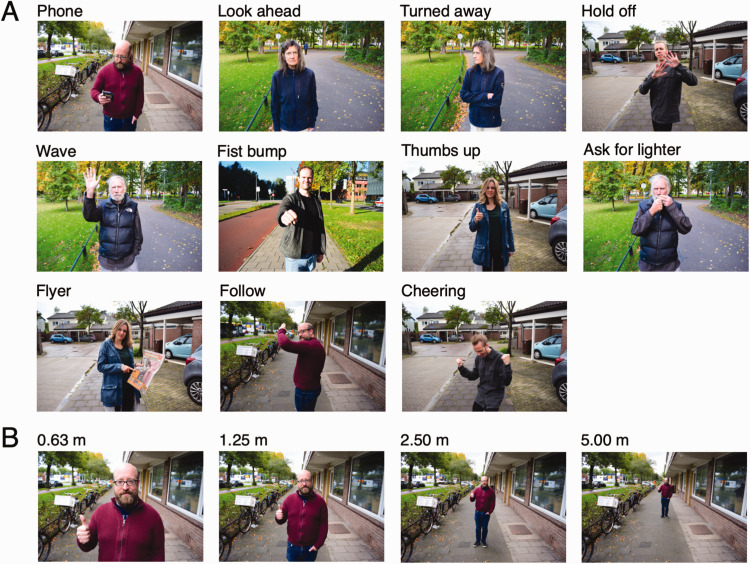
Example photographs used in the experiment. A: Example photographs for each behavior. Each behavior is depicted at the 1.25 m camera-actor distance such that the behavior is clearly visible. Six out of 11 actors are shown, and every location occurs at least once. B: Example photographs for the same behavior, actor, and location at each of the four camera-actor distances.

Pictures were shot using a Nikon D5300 DSLR camera with an 18-mm lens (70° by 50° field of view) at four camera-actor distances: 0.63, 1.25, 2.50, and 5.00 m, yielding a close-up, upper body shot, full body shot, and long shot, respectively. The camera was positioned at eye-height and the angle with respect to the street was adjusted for each walker such that the behaviors were clearly visible even at the shortest distance. Note that the actor was moved with respect to the camera as opposed to cropping and scaling the long shot, as the latter method would change what was visible in the background for each distance. Actors are portrayed in a suburban environment, on a sidewalk next to a street or houses, or in a park. A minimum of two actors were shot at each location (three actors at one location), such that actor identity was not uniquely coupled to a particular background. The stimulus set thus comprised 44 photographs per actor (each actor depicting 11 behaviors at 4 distances) and 484 photographs in total. Figure 1 depicts example photographs for each behavior.

### Procedure

The experiment was conducted online using Gorilla (Anwyl-Irvine, Massonnié, Flitton, Kirkham, & Evershed, 2020). In Gorilla, we restricted the experiment to desktop computers such that stimuli were presented on a sufficiently large display. Thus, Gorilla prohibited the experiment from being conducted on phones or tablets. First, an information letter was shown after which the participant gave informed consent. The experiment then began with an instruction screen stating:

You will be shown photographs very briefly. Each photograph contains a person. You need to judge what the person is doing and how you might respond (in English or Dutch). Before each photograph, you will see a dot. Please look at the dot and press the SPACEBAR. The dot will change color briefly before the photograph is flashed. Click the button below to get started.

**Figure 2.**
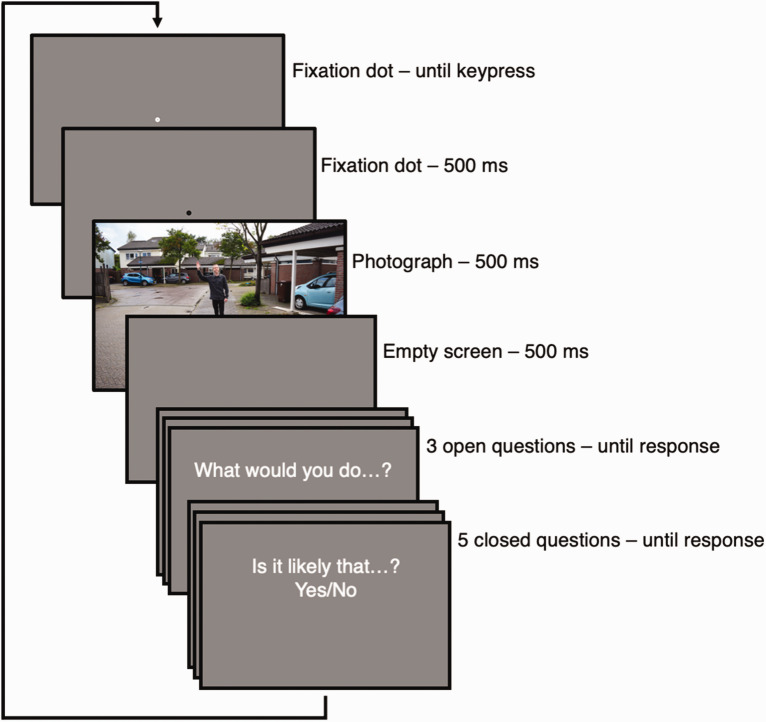
Trial structure of Experiment 1.

Once the participant commenced, 2 practice trials and 11 experimental trials were presented (see Figure 2 for the trial structure). The practice trials contained two photographs not otherwise included in the stimulus set and were the same for each participant. The experimental trials contained 11 photographs from the full set of 484 photographs. These were chosen pseudorandomly such that each behavior and each actor was shown only once per participant and each distance was shown a maximum of three times. The total set of 484 photographs was thus divided over 44 participants. The remaining eight participants were randomly presented with one of the 44 possible combinations of experimental trials. The background color for all screens was gray (HTML color code #888888).

The presentation duration for the photograph was 500 ms, based on previous research of perception “at a glance” (Fei-Fei et al., 2007) and pilot experiments. At this duration, participants’ viewing behavior is limited to about two fixations. Moreover, slight variation in the timing of the online experiment (e.g., one or two frames at 60 Hz) are within 10% at 500 ms, while it may amount to upwards of 33% at shorter presentation durations (e.g., 100 ms), which we deemed unwanted.

On each trial, the participant was asked three open questions and five closed questions. The open questions were “what would you do in this situation?”, “how did you decide what you would do?”, and “what stood out most in this photograph?”. These were meant to elicit a subjective description of what the participant perceived and how (s)he might respond in such a situation. Two of the closed questions were meant to quantify whether one perceived a situation as inviting interaction. These were “Is it likely that this person would interact with you?” (yes/no) and “How likely is the person to interact with you?” (rating scale: –3, *not at all*; 0, *neutral*; 3, *very likely*). These five questions combined allow a qualitative and quantitative description of the perception of invitations or intentions to interact.

The three remaining closed questions were included for various reasons. First, we wanted to know whether participants took the experiment seriously. For this, we asked the following question: “Was the person male or female?” (male/female). Assuming that this was an easy task to do, we could exclude participants were they to make a substantial number of errors. Second, we wanted to know what role our actors might play in being perceived as inviting interaction. We reasoned that actor familiarity and perceived friendliness might play a role. We therefore asked “How friendly would you rate this person?” (–3, *not at all*; 0, *neutral*; 3, *very friendly*) and “Do you know the person?” (yes/no). Note that for the rating scales, labels were only given for the values –3, 0, and 3. The full scale and the values were visible to the participant.

The experiment was found during piloting to take about 15–20 minutes to complete.

### Qualitative Analyses

A coding scheme was developed to manually annotate the answers to the three open questions. The goal of the coding scheme was to summarize the answers of multiple participants into a coherent set of answers. For example, for the question “what would you do in this situation?”, we grouped answers that reflected a similar strategy. The same principle was applied for the answers to the questions “how did you decide what you would do?” and “what stood out most in this photograph?”. As the coding scheme is an important result in itself, it is presented in the Results section. Here we outline briefly how the coding scheme was developed.

The coding scheme was drafted by author RH and refined through iterated discussions with authors IH, AD and JK. The revised scheme was then used to annotate a subset of answers (i.e., no more than 30 answers randomly drawn from the first few participants). All problem cases were discussed. The conclusions of these discussions were used to revise and clarify the coding scheme further.

The coding scheme was hierarchical, with possible subcategories to an overarching category. For each answer, multiple categories could be coded. All answers were annotated according to the scheme by five coders (four of the authors [RH, AD, JK and IH] and one coder naive to the purpose of this study). An odd number of coders was used so that a majority rule could be applied to determine the final codes for each answer. The coding was conducted using a custom point-and-click interface built in MATLAB (The Mathworks Inc.).

### Quantitative Analyses

For all quantitative analyses we used nonparametric bootstrapping with the Harrell-Davis estimator to compute medians and 95% confidence intervals. This is implemented in the MATLAB-function *decilespbci* provided by Rousselet et al. (2017). The number of bootstrap samples was set to the default value of 2000. We supplement our in-depth descriptions with Bayesian analyses conducted in JASP 0.14.1 (JASP Team 2020) where appropriate. For the Bayesian analyses, we use the notations for Bayes Factors (*BF_m_*, *BF*_10_, and *BF*_01_) as implemented in JASP. Briefly, the *BF_m_* quantifies the change from prior to posterior odds of a particular model. The *BF*_10_ represents the ratio between the likelihood of the alternative hypothesis given the data and the likelihood of the null hypothesis given the data. The *BF*_01_ represents the inverse of the *BF*_10_. For details, we refer the reader to the JASP website at https://jasp-stats.org/. For interpretation of the values, see for example, Table 1 in Schönbrodt and Wagenmakers (2018).

**Table 1.** Coding Scheme for the Three Open Questions.

Question	Coding categories	Interrater agreement
What would you do?	Respond nonverbally to actor	0.91
	*Make facial expressions*	*0.92*
	*Look or watch*	*0.86*
	*Make gesture or respond physically*	*0.89*
	*Take flyer*	*0.55*
	Adjust trajectory	0.74
	*Neutral connotation (make room, let pass)*	*0.47*
	*Negative connotation (back off, cross street)*	*0.69*
	*Follow*	*0.83*
	Continue walking (walk by, keep going)	0.75
	Ask from actor	0.88
	Speak to actor	0.86
	Listen to actor	0.82
	Be emotional, surprised, confused, etc.	0.62
	Do nothing, wait, or no decisive action	0.75
How did you decide?	Based on actor	0.09 (0.75)
	*Familiarity (I know this person)*	*0.73 (0.74)*
	*Random or unknown person*	*–0.06 (0.68)*
	Based on action	0.68
	*Looking behavior*	*0.80*
	*Facial expression*	*0.78*
	*Gesture*	*0.73*
	*Body posture*	*0.65*
	*Assumed intention (she wants to smoke)*	*0.43*
	*Empathic judgement (he looked friendly)*	*0.54*
	Based on object of action (absorbed in phone)	0.70
	Other arguments	0.64
	*Context (it was in a park)*	*0.60*
	*Instinct, feels normal, it seems natural*	*0.64*
What stood out most?	Actor	0.62
	*Body (big man, tall person)*	*0.22*
	*Hands*	*0.62*
	*Face or head (hair, make-up, familiar face)*	*0.82*
	*Gender (the man, the woman)*	*0.89*
	*Clothing*	*0.69*
	Action	0.84
	*Looking behavior*	*0.88*
	*Facial expression*	*0.83*
	*Gesture*	*0.72*
	*Body posture or body language*	*0.74*
	*Empathic judgement (the friendly girl)*	*0.52*
	Object of action (phone, flyer)	0.93
	Context (surroundings)	0.77

*Note.* Indented italicized lines indicate subcategories belonging to the overarching categories. For the purpose of clarifying some coding categories, hypothetical responses are given in parentheses. The measure for interrater agreement is Krippendorff’s alpha (Hayes & Krippendorff, 2007). The interrater agreement given in parentheses is computed for 4 out of 5 coders (see description in main text).

## Results

### Experimental Quality

Due to the online nature of the experiment, each participant conducted the experiment on a different setup. We therefore investigated the following aspects relating to the quality of the experimental procedure: (1) the size of the stimulus display, (2) whether all pictures were presented, (3) the duration of the sub-second displays (see Figure 2), and (4) the number of errors in identification of the actor gender. Aspects 1 and 3 were reported by Gorilla. Aspects 2 and 4 were derived from the participants’ answers to the open and closed questions.

The median viewport (the part of the screen available for stimulus presentation) height was found to be 843.5 pixels (sd= 167.7 pixels) and the median viewport width was 1536 pixels (sd= 332.6 pixels).

In 15 out of the total 572 trials (2.6%), the picture did not load. This occurred 8 times for one participant, 3 for another, and once for four additional participants. These trials were excluded from further analysis. The median duration of the sub-second displays as reported by Gorilla was 500 ms (sd= 1.7 ms, range 492–524 ms) for the pre-image fixation dot, 500 ms (sd= 1.6 ms, range 492–510 ms) for the image display, and 500 ms (sd= 1.4 ms, range 493–509 ms) for the postimage blank screen. Thus, although sometimes a picture would not load, it seemed that the duration of the image displays (and preceding and subsequent displays) was close to the intended duration of 500 ms.

Eleven errors were made in identifying the gender of the actor. This did not occur more than once for any participant, and always occurred for one of two female actors, one elderly woman, and one young woman wearing overalls. Due to the low occurrence of misidentifications, we concluded that all participants conducted the experiment seriously.

### Qualitative Assessment

The qualitative answers given by the participants were used to assess what was perceived in terms of the potential for interaction and how one might respond in such a situation. In order to group the answers of multiple participants, we developed a coding scheme to analyze the responses. We first present the coding scheme and interrater agreement. Hereafter, we report what types of responses were most common, and how some of the responses depended on the behavior depicted in the photographs. Finally, we discuss example responses that stood out or are of specific interest to our research question.

#### Coding Scheme

The coding scheme arrived at through iterated discussions is presented in Table 1. Eight main categories were identified for the answers to the question “What would you do in this situation?” For the categories “Respond nonverbally” and “Adjust trajectory” several subcategories were identified. For the questions “How did you decide what you would do?” and “What stood out most in this photograph?” four main categories were identified: actor, action, object of action, and context or other arguments.

#### Interrater Agreement

The interrater agreement for the five coders was estimated using Krippendorff’s alpha (Hayes & Krippendorff, 2007). The values are presented in Table 1. Interrater agreement for the overarching categories ranged from 0.62 to 0.91 for the first question (“What would you do…?”), from 0.09 to 0.70 for the second question (“How did you decide…?”) and from 0.62 to 0.93 for the third question (“What stood out most…?”). The low interrater agreement of 0.09 for the “Based on actor” category for the second question stood out. Upon investigation of the codings from the individual coders, it turned out that one coder coded the “Random or unknown person”-subcategory substantially different from the other four coders. Note that this was not the naive coder, but one of the authors. Interrater agreement for the other four coders was 0.68 for this subcategory, and 0.75 for the overarching “Based on actor”-category. Interrater agreement for the subcategories were sometimes lower than 0.6, often for subcategories that did not occur often.

We concluded that there was substantial interrater agreement, particularly for the first and third question. We determined the final set of codings by taking a majority rule for the five coders.

#### Response Frequencies

**Figure 3.**
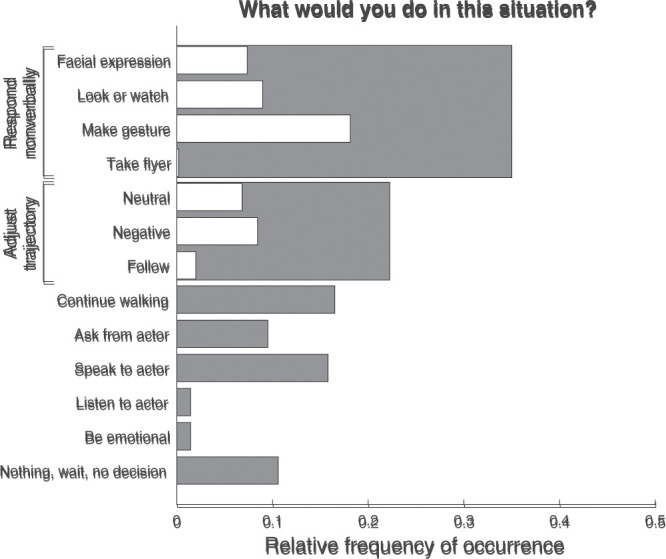
Relative frequency of occurrence for the various response types to the question “What would you do in this situation?”. The relative frequency of occurrence represents the proportion of trials on which a particular type of response was given. For categories with multiple subcategories, the gray bars indicate the total relative frequency of occurrence for overarching categories, that is, when at least one subcategory (white bars) or only the overarching category was coded. As multiple categories may be coded for each response, the relative frequencies of the subcategories do not sum to the relative frequency of the overarching category. Neither do the relative frequencies of the overarching categories sum to one. Note that the “Take flyer” response is close to zero.

Figure 3 depicts the relative frequency that each type of response occurred for the question “What would you do in this situation?”, calculated as the number of times a response category was coded divided by the total number of responses (i.e., trials). For the vast majority of responses, a clear action was formulated. In only 11% of all trials did a participant decide to wait, do nothing, or took no clear decision. Regarding the latter, responses were sometimes given in which the decision depended on the conditions under which the decision would have to be made in the actual situation, for example, if X would happen, I would do Y or if I knew him, I would do Z. Such responses were ambiguous in the sense that the coder could not determine what action would be taken.

Nonverbal responses towards the actor or based on what the actor portrayed were most common, occurring for 35% of all trials. A nonverbal response could be making facial expressions, looking in a particular manner, making or returning a gesture, or accepting a flyer from the actor. Responses in which the trajectory would somehow be adjusted were also quite common, occurring on 22% of all trials. The participants indicated that they would continue walking, ask something from the actor, or speak to the actor in 10% to 17% of all trials.

**Figure 4.**
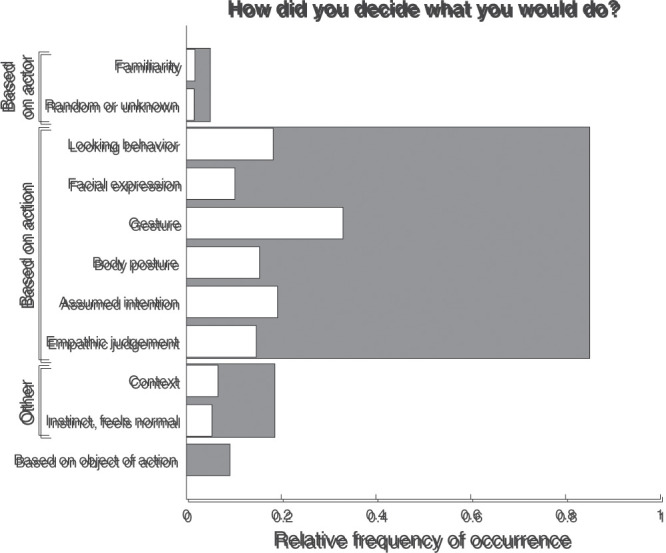
Relative frequency of occurrence for the various response types to the question “How did you decide what you would do?”. The relative frequency of occurrence represents the proportion of trials on which a particular type of response was given. For categories with multiple subcategories, the gray bars indicate the total relative frequency of occurrence for overarching categories, that is, when at least one subcategory (white bars) or only the overarching category was coded. As multiple categories may be coded for each response, the relative frequencies of the subcategories do not sum to the relative frequency of the overarching category. Neither do the relative frequencies of the overarching categories sum to one.

Figure 4 depicts the frequency that each type of response occurred for the question “How did you decide what you would do?”. As is visible, in almost all cases (85% of all trials), the decision was based partly on the action depicted by the actor in the photograph. This could be based on the looking direction, facial expression, gesture, or posture depicted, but also on an assumption about the actor’s intention or an empathic judgement about the actor. In 19% of all trials, other arguments were given for the participant’s decision, with reference to, for example, the context (7% of all trials), or to instinct or what feels normal (5% of all trials). In 9% of all trials, the object of the action (e.g., the phone or flyer) was part of the argumentation. In even fewer trials was the decision based on the actor, for example because (s)he looked familiar, or because it is what one would do when encountering a “random” person.

**Figure 5.**
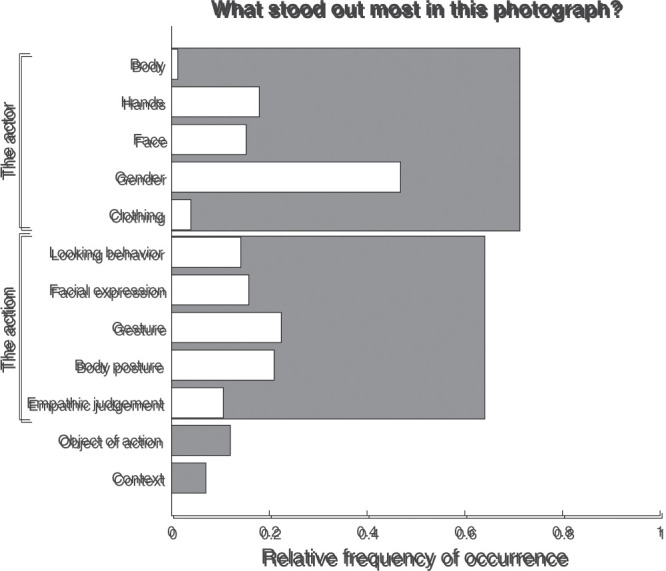
Relative frequency of occurrence for the various response types to the question “What stood out most in this photograph?”. The relative frequency of occurrence represents the proportion of trials on which a particular type of response was given. For categories with multiple subcategories, the gray bars indicate the total relative frequency of occurrence for overarching categories, that is, when at least one subcategory (white bars) or only the overarching category was coded. As multiple categories may be coded for each response, the relative frequencies of the subcategories do not sum to the relative frequency of the overarching category. Neither do the relative frequencies of the overarching categories sum to one.

Figure 5 depicts the frequency that each type of response occurred for the question “What stood out most in this photograph?”. In a large proportion of the cases (71% of all trials), some aspect of the actor stood out, such as the gender, clothing, face, hands, or body. In a slightly smaller number of cases (64% of all trials), an aspect of the action stood out, such as the looking behavior, facial expression, gesture, body posture, or an empathic judgement about the depicted action. The object of the action or the context were mentioned in 12% and 7% of all trials, respectively. Note, however, that not all actions had an object it was directed at.

Based on these findings, we conclude that participants perceived the gist (i.e., the general setting and potential action of the other person) of the photographs at a glance and formulated clear responses to the situations depicted in the photographs. Although aspects of both the actor and action stood out in most cases, the decision what to do seemed based primarily on aspects of the depicted action. However, objects at which actions are directed, actor familiarity, context, social conventions or instinct are potential other reasons or cues for what one might do in a given situation. These are likewise perceived at a glance and subjectively available to the observer.

#### Response Frequencies as a Function of Behavior

One wonders how the responses given to the three open questions in this experiment depended on the behavior depicted in the photograph. However, not all response types lend themselves equally to separating by behavior. For example, some responses rarely occurred (<20% of all trials) or occurred very often (>80%), such as the “based on actor” and “based on action” categories for the question “How did you decide what you would do?”. In this case, there is little room for variability across the 11 behaviors. Another example is that several response types could only occur for certain behaviors, such as the “object of action”-category for the Phone and Flyer behaviors. Separating this particular response type by behavior is therefore not informative. We therefore restrict our analysis to two cases.

**Figure 6.**
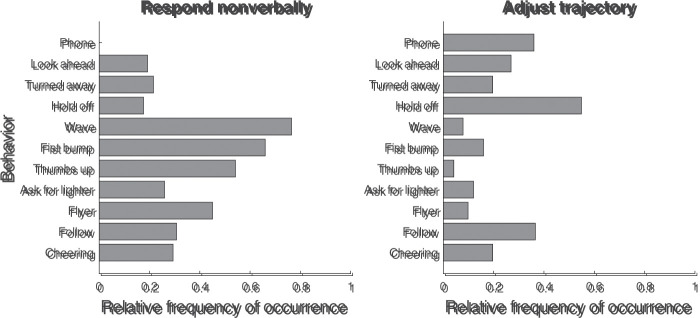
Relative frequency of occurrence for the “respond nonverbally” (left panel) and “adjust trajectory” (right panel) response types to the question “What would you do in this situation?”, separated by behavior. The relative frequency of occurrence represents the proportion of trials on which a particular type of response was given.

The first interesting case consists of the “respond nonverbally” and “adjust trajectory” response categories for the question “What would you do in this situation?”, which were coded on 20% to 40% of all trials. Separating these categories by behavior allows us to answer the question of how the participants’ likely response in a situation depended on the behavior of the actor. Figure 6 depicts the relative frequency of occurrence for the “respond nonverbally” and “adjust trajectory” categories as a function of behavior depicted in the photograph. As can be seen in the left panel, nonverbal responses occurred most often for the Wave, Fist bump, Thumbs up, and Flyer behaviors. Nonverbal responses were not mentioned for the Phone behavior, and occurred little for the Look Ahead, Turned Away and Hold off behaviors. Conversely, adjustments of the walking trajectory (right panel in Figure 6) were most often mentioned for the Hold off, Follow, Phone and Look ahead behaviors, while occurring little for the Wave, Fist bump, Thumbs up, Ask for lighter and Flyer behaviors.

Statistical analyses using Bayesian contingency tables in JASP supported the notion that the responses depended on the depicted behavior. The Bayes factor (*BF*_10_) for the hypothesis that the proportion of trials in which a nonverbal response was mentioned was not uniform across the behaviors was $1.6\times 10^{21}$. The Bayes factor for the hypothesis that the proportion of trials in which an adjustment of the trajectory was mentioned was not uniform across the behaviors was $5.5\times 10^{8}$. Thus, we conclude that the participants’ responses about what they would do depended on the behavior depicted in the photograph.

**Figure 7.**
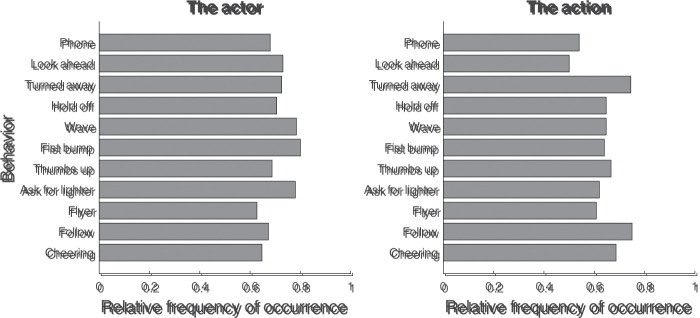
Relative frequency of occurrence for the “actor” (left panel) and “action” (right panel) response types to the question “What stood out most in this photograph?”, separated by behavior. The relative frequency of occurrence represents the proportion of trials on which a particular type of response was given.

The second interesting case consists of the “actor” and “action” response categories for the question “What stood out most in this photograph?”, occurring on 60% to 80% of all trials. Separating these categories by behavior allows to answer the question of whether what stood out to the participant depended on what the actor was doing. Figure 7 depicts the relative frequency of occurrence for the “actor” (left panel) and “action” (right panel) response categories as a function of the behavior depicted in the photograph. As can be seen, there does not seem to be a clear pattern that either the actor or action stood out more or less for some behaviors than for others.

Statistical analyses using Bayesian contingency tables in JASP supported the notion that what stood out most did not depend on the depicted behavior. The Bayes factors (*BF*_01_) for the null hypotheses that the proportion of trials in which the actor or action were mentioned was uniform across behaviors were 7371 and 1315, respectively. Thus, we conclude that whether an aspect of the actor or action stood out in a photograph did not depend on the behavior depicted.

#### Response Frequencies as a Function of Distance

Similar to the relation between participants’ responses and the behavior depicted in the photograph, one may wonder how the responses depended on the camera-actor distance in the photograph. Bayesian contingency tables revealed that for all of the four response categories examined above (“respond nonverbally,” “adjust trajectory,” “actor,” and “action”), there was no relation between the relative frequency of occurrence and the camera-actor distance. Bayes factors (*BF*_01_) in support of the null hypothesis that the proportion of trials was uniform across distance were 90 for the “nonverbal response” category, 49 for the “adjust trajectory” category, 51 for the “actor” category, and 139 for the “action” category. The corresponding proportions are reported in Table 2. Thus, whether participants were likely to respond nonverbally or adjust their trajectory did not depend on the camera-actor distance. Neither did aspects of the actor or action stand out more or less depending on the camera-actor distance.

**Table 2.** Relative frequencies of occurrence for the “respond nonverbally” and “adjust trajectory” response types for the question “What would you do in this situation?” and the “actor,” and “action” response types for the question “What stood out most in this photograph?”, separated by distance.

Category	0.63 m	1.25 m	2.50 m	5.00 m
Respond nonverbally	0.34	0.38	0.38	0.31
Adjust trajectory	0.21	0.19	0.23	0.26
Actor	0.73	0.76	0.68	0.68
Action	0.62	0.63	0.68	0.64

*Note.* The relative frequency of occurrence represents the proportion of trials on which a particular type of response was given.

#### Example Responses

A number of responses stood out during the annotation that were not captured in the coding scheme, but are of interest, for example, in light of previous research or given the coronavirus disease (COVID-19, henceforth COVID for brevity) pandemic taking place at the time of data collection.

Several participants mentioned the COVID-pandemic, either implicitly or explicitly. One participant, for example, mentioned thrice that (s)he would take a step back while referring to COVID. Interestingly, this occurred only for trials in which the depicted distance was 0.63 or 1.25 m, that is, below the 1.5 m distance regulation in place in the Netherlands during data collection. Another participant responded that (s)he would move more aside to maintain distance given the current COVID situation. Yet another mentioned that (s)he might have been coughing too close to the actor (for the “Hold off” behavior). Finally, one participant mentioned that (s)he would touch the elbow of the actor with her elbow, seemingly interpreting the “Follow”-behavior as an invitation for a COVID-proof “handshake.” Thus, we observed an effect of the COVID-regulations on some participants’ responses of how they would react in a depicted situation and why they would do so.

Another set of intriguing responses was given for the “Turned away” behavior. Some participants mentioned that they would look in the same direction the person is looking in. On two occasions, the participant explicitly mentioned first passing the person, after which they would look in the direction the person was looking in. This matches well with studies by Gallup et al. (Gallup, Chong, et al., 2012; Gallup, Hale, et al., 2012), who have shown that people tend to look where others in their proximity look, but that this occurs more often when others are oriented away from the person. At least some of our participants were thus able to verbalize this phenomenon of walking past another person before following their looking direction.

Finally, one participant gave two interesting answers to the question “How did you decide…?” Once, (s)he answered “by looking at the scene shown and not having anything to do with it” and once “by having looked at the photo”. This participant verbalized that (s)he was in fact not present in the actual scene, but merely observing a photograph. This is interesting, as in essence we asked our participants to place themselves in a different world (cf. Bracker, 2017). What is perhaps more striking is that only two responses were of this kind. The vast majority of participants described what they might do based on the depicted actor and actor behavior. This suggests that participants were perfectly able to imagine themselves being in the depicted situation and verbalizing potential actions accordingly.

### Quantitative Assessment

The closed questions were used to quantify whether one perceived a situation as inviting interaction. First, we investigated the likeliness to interact as a function of the depicted behavior, distance, and combination of the two. Second, we investigated the role of actor and participant gender. Finally, we investigated the relation between actor familiarity, perceived friendliness, and the likeliness to interact.

#### Likeliness to Interact as a Function of Behavior

**Figure 8.**
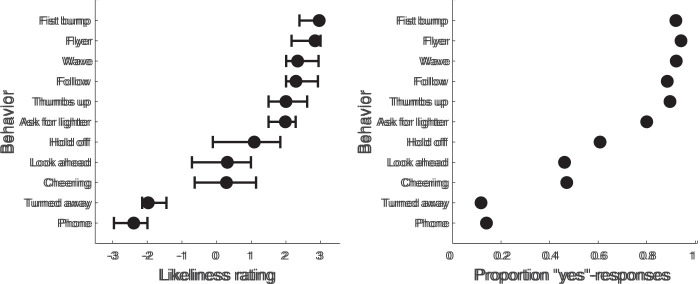
Likeliness to interact as a function of actor behavior. The left panel depicts the likeliness to interact ratings. Black markers and lines indicate medians and 95% confidence intervals acquired through nonparametric bootstrapping. The number of bootstrap samples was 2000. The right panel depicts the proportion of “yes”-responses to the question “Is it likely that this person would interact with you?”. For both panels, the behaviors are sorted by the median likeliness to interact ratings. Each participant contributed one trial to each behavior (barring excluded trials, see Experimental quality section).

The left panel in Figure 8 depicts the likeliness to interact ratings (ranging from –3 [*not at all likely*] to 3 [*very likely*]) as a function of the behavior depicted in the image. As is obvious from this figure, some behaviors yielded high likeliness to interact ratings (e.g., the fist bump, showing a flyer, waving, or signing to follow), whereas others yielded low likeliness to interact ratings (an actor looking at their phone, or turned away). The three remaining behaviors (actor trying to maintain distance, looking ahead, or cheering) were rated somewhere in between, with larger confidence intervals. This indicates there was more variability between participants in how these behaviors where apperceived. Note that the pattern of results is similar for the binary question of whether it is likely that the person would interact (see Figure 8, right panel). We conclude that the likeliness to interact clearly depends on the depicted behavior.

#### Likeliness to Interact as a Function of Distance

**Figure 9.**
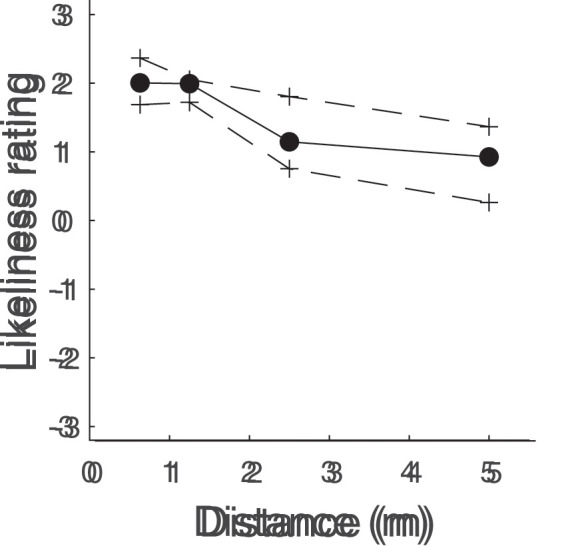
Likeliness to interact ratings as a function of the four camera-actor distances used for the set of images. Black dots and solid lines indicate bootstrapped medians and crosses and dashed lines indicate bootstrapped 95% confidence intervals. Each participant contributed three trials for three distances, and two trials for one distance (barring excluded trials, see Experimental quality section).

Figure 9 depicts the likeliness to interact ratings as a function of the four camera-actor distances used for the set of images. As can be seen, likeliness to interact ratings were somewhat higher for the shorter distances (0.63 and 1.25 m) than for the longer distances (2.50 and 5.00 m). This indicates that participants deemed interaction to be more likely at shorter distances than at longer distances. However, it may be the case that this pattern is not identical for every behavior.

#### Likeliness to Interact as a Function of Behavior and Distance

**Figure 10.**
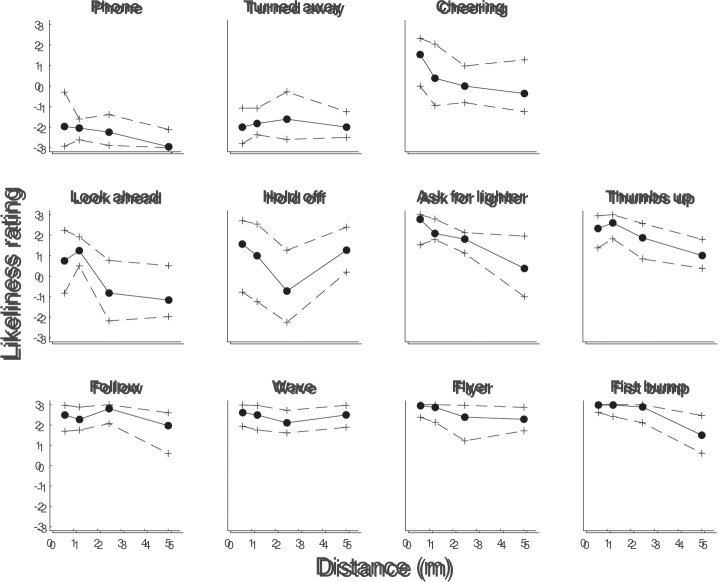
Likeliness to interact ratings as a function of the four camera-actor distances used for the set of images, separated for the 11 behaviors. Black dots and solid lines indicate bootstrapped medians and crosses and dashed lines indicate bootstrapped 95% confidence intervals. Behaviors are sorted according to the overall median likeliness to interact ratings (see Figure 8). Each participant contributed a maximum of one trial for each behavior-distance combination. The number of participants per computed median ranged from 11 to 15.

Figure 10 depicts the likeliness to interact ratings as a function of the four camera-actor distances used for the set of images, separated for each of the 11 behaviors. Three patterns can be identified. There are those behaviors for which likeliness to interact does not seem to depend on the camera-actor distance, for example, the Phone, Turned away, Follow, Wave, and Flyer behaviors. For these, the likeliness to interact was consistently low or high. For the Ask for lighter, Thumbs up, and the Fist bump behaviors, the likeliness to interact ratings seems to be lower for the longer distances. Interestingly, these three behaviors have in common that they contain a gesture directed at the camera. A potential explanation for the lower likeliness to interact ratings for longer distances could be that these gestures could be identified less well at these distances.

The final three behaviors (Cheering, Look ahead, and Hold off) had a markedly different pattern and larger confidence intervals (more between-participant variability). For the Cheering behavior, the likeliness to interact rating was substantially higher for the shortest distance than for the three other distances. For the Look ahead behavior there seems to be a difference in likeliness to interact between the two shortest and two longest distances. For the Hold off behavior, there seems to be an interesting dip in the likeliness to interact ratings for the 2.50 m distance, although the confidence intervals are large.

We summarized our findings statistically using a Bayesian analysis of variance (ANOVA). The likeliness to interact ratings were used as the dependent variable, behavior and distance as fixed factors, and participant as a random factor. This revealed that the model with the behavior and distance terms was best supported by the data (*BF_m_* = 75), that is, better than models with only the behavior or distance as fixed factors, the model including the interaction term (behavior × distance), or the null model including only the participant as a random factor. The best fitting model was at least 19 times as likely as any other model (i.e., *BF*_01_ as reported by JASP).

Based on our findings and the statistical analysis, we conclude that the perceived likeliness to interact depends on both the depicted behavior and the distance to the actor. Patterns of likeliness to interact as a function of distance seemed to differ between behaviors based on the bootstrapped medians and 95% confidence intervals. Yet, this was not supported by the statistical analysis, perhaps given the relatively small number of observations (11–15) per behavior-distance combination.

#### Actor and Participant Gender

As gender differences have been observed for gist perception of social scenes by Vanmarcke and Wagemans (2015), we checked the relation between the likeliness to interact ratings and actor and participant gender. For this, we computed the average likeliness to interact ratings for male and female actors per participant. We then conducted a Bayesian repeated measures ANOVA on these average likeliness to interact ratings with actor gender as a repeated-measures factor and participant gender as a between-subjects factor. The null model was best supported by the data ($BF_{m}=3.6$), and 2.2 times as likely as any other model (i.e., *BF*_01_ as reported by JASP). Thus, neither actor nor participant gender seemed to be strongly related to the likeliness to interact ratings, and we do not consider gender differences further.

#### Actor Familiarity, Perceived Friendliness, and Likeliness to Interact

We investigated what role our actors might have played in being perceived as inviting interaction. For this, we related actor familiarity with perceived friendliness and the likeliness to interact. Actors were recognized on 15.6% of all trials, ranging between 0% and 41.2% for the various actors. Perhaps unsurprisingly, the first author was recognized most often. The median perceived friendliness for known actors (median 1.83, 95% CI 1.16–2.03) was higher than for unknown actors (median 0.917, 95% CI 0.26–1.00), as indicated by the nonoverlapping confidence intervals. However, it did not seem that actor familiarity was related to the likeliness to interact ratings: The median likeliness to interact rating for known actors was 1.54 (95% CI 0.75–2.11), whereas the median likeliness to interact rating for unknown actors was 1.80 (95% CI 1.11–2.00). The 95% confidence intervals overlapped substantially.

**Figure 11.**
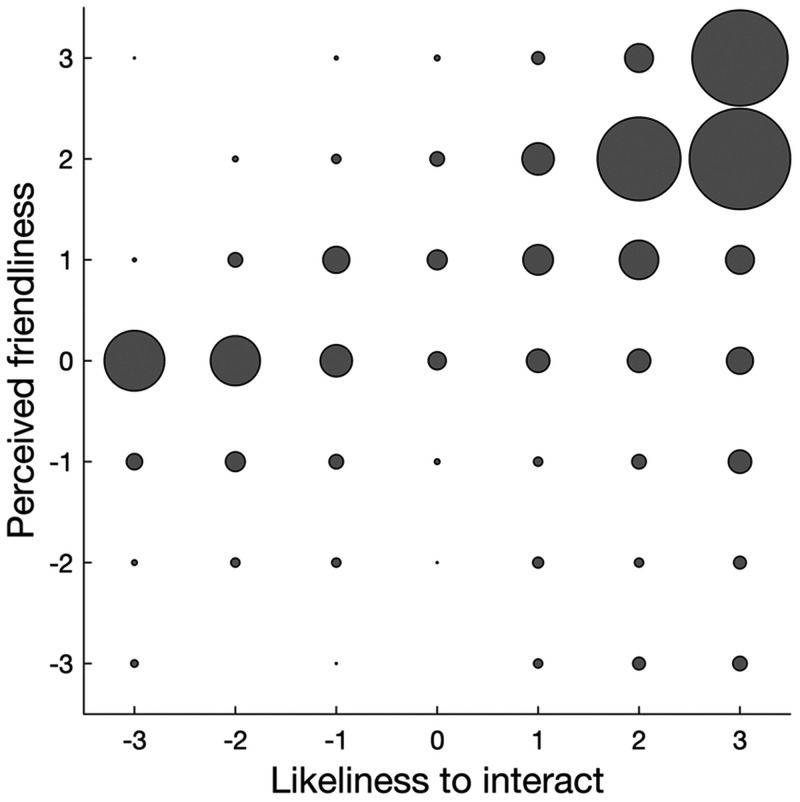
Relation between perceived friendliness and likeliness to interact ratings. The radius of each bubble represents the number of occurrences for the combination of friendliness and likeness to interact ratings. The maximum number of occurrences was 57 for a likeliness to interact rating of 3 and friendliness rating of 2.

The relation between the perceived friendliness and likeliness to interact ratings is depicted in Figure 11. As visible, higher likeliness to interact ratings were associated with higher friendliness ratings. The Pearson correlation coefficient was 0.38, and the corresponding Bayes Factor for there being a correlation as opposed to no correlation was $2.1\times 10^{17}$.

We conclude that actor familiarity was related to perceived friendliness, but not the likeliness to interact, and that perceived friendliness and likeliness to interact were substantially correlated.

## Discussion

We investigated what humans perceive in terms of invitations to interact at a glance, and how they might respond in such situations. For this, we briefly presented participants with photographs depicting 11 prototypical invitations and noninvitations to interact at four camera-actor distances. We elicited both qualitative and quantitative responses from the participants to answer our research question.

Our analysis of the qualitative responses revealed that participants were able to formulate clear responses about what they might do for each situation. What the participants would do depended on the behavior depicted in the photograph, but not the camera-actor distance. Whether aspects of the actor or action stood out in the photograph did not depend on either the depicted behavior or the camera-actor distance. The decision of what participants would do in a given situation seemed based primarily on aspects of the behavior depicted in the photograph. However, the object of the action, actor familiarity, context, social conventions or instinct were other cues or reasons participants mentioned for what they might do in a given situation. Based on these findings, we conclude that participants perceived the gist—that is, the general setting and potential action of the other person—of the photographs. Moreover, various aspects of the actor, action, and context depicted in the photograph are subjectively available at a glance.

The description of our qualitative findings are interesting in the context of previous research on scene perception at a glance. Fei-Fei et al. (2007) collected free-form descriptions from participants of scenes presented for various durations. They reported that “a rich collection of perceptual attributes is represented and rises to conscious memory within a single fixation” and that “more cognitive appraisals of the event–such as social interaction and sports events–can be recognized effortlessly” (p. 22). We corroborate the latter statement and add that humans can verbalize meaningful responses about how they might act based on a glance of such events. The responses given by our participants moreover suggest that they were perfectly capable of imagining themselves in the depicted situation (cf. “presence” in IJsselsteijn et al., 2000) and were intimately familiar with the depicted behaviors (cf. Shklovsky, 1917). Participants not only described what the actor in the photograph was doing, but also what the actor would or might do if the situation were to continue from what was depicted (cf. the “Assumed intention”-category in Figure 4). Clearly, the photographs suggested “happenings” that extend in lived time (Koenderink et al., 2020). Thus, in principle still images do not preclude one from studying actions or events in scene perception (cf. Võ, 2021, pp. 16–17 and McArthur and Baron, 1983, p. 216). That being said, one wonders how the perception of invitations and noninvitations at a glance differ in virtual or real environments. Obviously for the latter one would have to solve the technical problem of presenting a real environment at a glance.

Our analysis of the quantitative responses revealed that whether participants expected interaction to occur depended on both the depicted behavior and the camera-actor distance. The fact that the likeliness to interact ratings depended on the camera-actor distance is interesting, given that the camera-actor distance was not related to what participants would do. Thus, whether one perceives interaction to be likely in a situation is not equivalent to what one expects to do in a situation. Two interesting additional patterns stood out regarding the likeliness to interact ratings. For the thumbs up, fist bump and ask-for-lighter gestures, the likeliness to interact ratings were generally high, but they seemed somewhat lower for the longer camera-actor distance. Perhaps these gestures could be identified less well for longer camera-actor distances than they could be for shorter camera-actor distances. For three other behaviors (the Cheering, Look ahead, and Hold off behaviors), there seemed to be more interindividual variability in the likeliness to interact ratings than for other behaviors. These behaviors might have been most ambiguous in terms of what was happening or likely to happen.

Three additional findings were obtained from the quantitative responses. First, the perceived friendliness of the actor was correlated with the likeliness to interact ratings. Second, we found that participants rated actors they knew as more friendly. Third, actor familiarity was not related to the likeliness to interact ratings. Given the correlational nature of the first finding, we cannot conclude whether friendly actors are more likely to be interacted with, or whether actors that one foresees interacting with are deemed more friendly. The latter two findings suggest that participants were able to judge the photographs by what action was depicted by the actor regardless of whether they knew the actor.

As stated in the introduction, humans can perceive many cues that may be relevant to another’s invitation or intention to interact. This raises the question of whether the likeliness to interact ratings could be predicted by such single cues. Consider gaze direction as a potential cue. Sweeny and Whitney (2017) write that whether “a person is looking directly at you is […] a strong predictor that a social interaction may occur” (p. 67). If we compare this statement to our findings (in particular Figure 8), we see that the median likeliness to interact ratings are lowest for the three behaviors in which the actor does not look in the direction of the camera (Phone, Turned away, and Cheering). However, two other behaviors for which the actor does look in the direction of the camera (Look ahead and Hold off) score in the same range as the Cheering behavior. Thus, the gaze direction may predict the perceived likeliness to interact, although it clearly depends on what else the actor does.

One could consider the actor’s behavior to be a unique combination of various cues (e.g., body orientation, gaze direction, gestures, facial expression, the “observables” in Dijksterhuis & Bargh, 2001). In that trivial sense, the combination of cues predicts the perceived likeliness to interact. What is interesting, however, is whether certain behaviors exist that are Gestalt-like, in the sense that they are perceived as inviting interaction, whereas their “single cues” might not. Such a holistic way of viewing the problem may be useful for modeling encounters in social robotics, as opposed to focusing only on one or a few cues (as e.g., in Peters, 2005). If one were to pursue the topic from an inferential perspective, it would at least be interesting to determine the degree of the “mutual substitutability” among various cues (see Brunswik, 1955, p. 207).

There are also alternatives to a purely cue-based perspective on social perception. Dijksterhuis and Bargh (2001), for example, state that “we perceive more than is literally present. Apart from perceiving observables, we make trait inferences and activate social stereotypes.” They argue that these three components influence behavior through imitation. However, their model of social perception does not yield predictions about the phenomenology of what observers perceive in terms of invitations to interact.

A second alternative to consider is the ecological theory of perception (Gibson, 1979). Ecological theories of perception are often contrasted with the “information-processing approach [which] views perception as a process of inference” (McArthur & Baron, 1983, p. 234). McArthur and Baron argue that the notion of affordances is particularly useful for studying social perception: What does the environment afford the perceiver in terms of, for example, the potential for interaction? But what are these affordances? According to McArthur and Baron, they are “typically complex properties that have no one-to-one connection to the static, stimulus elements that are provided to perceivers in traditional research paradigms” (p. 234). However, the static stimuli we used in the present study clearly elicited responses about affordances (i.e., what an observer might act upon) even when viewed at a glance. In our view, the phenomenology of perceiving social scenes is best addressed from the perspective of meaning that is imposed by an observer rather than stimulus information in the world (see e.g., Koenderink, 2019). It is clear that substantial work needs to be done in order to develop a theory from which predictions can be derived about the phenomenology of social perception. The present study hopefully serves as a useful initial empirical basis.

A note on generalization is in order. The behaviors we used were selected for their prototypical nature in the context of the Netherlands (from which most, but not all, of our participants were recruited). The gestures (fist bump, thumbs up) or greetings (wave) are commonplace in the current Dutch context, but may not be so in other contexts or in the future (see e.g., Matsumoto, 2006, for a review on culture and nonverbal communication). (Technical) gesture use has changed throughout history (e.g., Bremmer & Roodenburg, 1991; Hall, 2004; Kendon, 1983), and greetings may differ in nonverbal (Patterson et al., 2007) or verbal (Pinto, 2008) content across cultures. In addition, cross-cultural differences in the perception of eye contact have been observed (Akechi et al., 2013). In fact, we directly observed the effect of the “zeitgeist” in the participants’ responses. At the time of data collection, regulations against the spread of a coronavirus were in place, regulating, for example, interpersonal distances in- and outdoors. This was evident in some of the participants’ answers about what they would do in a situation, or why they would do so. In one case, a beckoning gesture was perceived as an invitation to bump elbows, that is, a COVID-proof “handshake.” What is perceived in a scene is thus observer-dependent, shaped at various timescales. The specific behaviors or gestures photographed for the present study are therefore not of primary interest. What is important is that participants recognized the various behaviors, and that different responses were formulated depending on those behaviors. We expect similar patterns to be observed for other culture-specific greetings or gestures.

In a similar vein, one may expect that our findings are specific to the social context depicted in the photographs, that is, an encounter with one other person carrying out some particular action at various distances. One may wonder whether the relation between camera-actor distance and the likeliness to interact ratings is modulated by the number of other people present in a scene, for example in dense crowds. Likewise, the context of an encounter in daylight may be quite different from an encounter at night in a poorly lit environment (see e.g., Boomsma & Steg, 2014, regarding the link between lighting level and perceived social safety). We expect that such differences will affect a person’s perceived likeliness to interact, as well as perhaps the aspects of the actor, action, and environment that stand out at a glance. We encourage future research to tackle these questions as they have clear applications in, for example, the design of public spaces.

In conclusion, we show that humans can perceive the gist of briefly presented photographs depicting various invitations and noninvitations to interact. Humans formulate clear responses of what they might do in those situations. A number of interesting follow-up questions come to mind. First, following Fei-Fei et al. (2007), one may wonder which behaviors are perceived and adequately responded to even under very brief presentation (<200 ms). Is it the case that some behaviors stand out almost regardless of presentation time? Or is there a minimum duration that a photograph must have been shown for it to be recognized and responded to? Second, one may wonder how snapshots of dynamic encounters are perceived (e.g., short videoclips). Perhaps the dynamic aspects may quickly disambiguate certain behaviors and lead to clearer approach or avoid responses. Third and finally, it may be expected that some behaviors containing gestures may not be perceived under degraded presentation as the gestures themselves become unrecognizable. The stimulus set used in the present study is made public^3^ for 9 out of 11 actors, which may be used to address some of these questions.

## Notes

1 Ensemble perception is thought to provide the basis for gist perception (Alvarez, 2011).

2 In fact, this is recognized as a limitation in research on the prediction of kicker direction (Diaz et al., 2012). Under normal circumstances there is feedback from goalkeeper to kicker and vice versa, which is not captured when one present videoclips of kickers to a noninvolved observer.

3 https://osf.io/8ecv6/.

## References

[bibr1-20416695211040237] Akechi H. Senju A. Uibo H. Kikuchi Y. Hasegawa T. Hietanen J. K. (2013). Attention to eye contact in the west and east: Autonomic responses and evaluative ratings. PLoS ONE, 8(3), e59312. 23516627 10.1371/journal.pone.0059312 PMC3596353

[bibr2-20416695211040237] Albertazzi L. (2013). Handbook of experimental phenomenology: Visual perception of shape, space and appearance. John Wiley & Sons: Chichester, UK.

[bibr3-20416695211040237] Alvarez G. A. (2011). Representing multiple objects as an ensemble enhances visual cognition. Trends in Cognitive Sciences, 15(3), 122–131. 21292539 10.1016/j.tics.2011.01.003

[bibr4-20416695211040237] Anstis S. (2018). The role of the pupil, corneal reflex, and iris in determining the perceived direction of gaze. i-Perception, 9(4), 1–4. 10.1177/2041669518765852 PMC6158516 30275943

[bibr1000-20416695211040237] Anwyl-Irvine, A. L., Massonnié, J., Flitton, A., Kirkham, N., & Evershed, J. K. (2020). Gorilla in our midst: An online behavioral experiment builder. *Behavior research methods*, *52*(1), 388–407, 10.3758/s13428-019-01237-x PMC7005094 31016684

[bibr5-20416695211040237] Berry D. S. Misovich S. J. (1994). Methodological approaches to the study of social event perception. Personality and Social Psychology Bulletin, 20(2), 139–152.

[bibr6-20416695211040237] Blake R. Shiffrar M. (2007). Perception of human motion. Annual Review of Psychology, 58(1), 47–73. 10.1146/annurev.psych.57.102904.190152 16903802

[bibr7-20416695211040237] Boomsma C. Steg L. (2014). Feeling safe in the dark: Examining the effect of entrapment, lighting levels, and gender on feelings of safety and lighting policy acceptability. Environment and Behavior, 46(2), 193–212.

[bibr8-20416695211040237] Bracker J. (2017). The borders of metalepses and the borders of the image. In Grabbe L. C. Rupert-Kruse P. Schmitz N. M. (Eds.), Bildverstehen. Spielarten und Ausprägungen der Verarbeitung multimodaler Bildmedien (pp. 93–109). Büchner-Verlag: Darmstadt.

[bibr9-20416695211040237] Bremmer J. Roodenburg H. (Eds.). (1991). A cultural history of gesture: From antiquity to the present day. Polity Press: Cambridge, UK.

[bibr10-20416695211040237] Brunswik E. (1955). Representative design and probabilistic theory in a functional psychology. Psychological Review, 62(3), 193–217. 10.1037/h0047470 14371898

[bibr11-20416695211040237] Cantor N. Mischel W. (1979). Prototypes in person perception. In *Advances in experimental social psychology* (Vol. 12, pp. 3–52). Elsevier.

[bibr12-20416695211040237] Diaz G. J. Fajen B. R. Phillips F. (2012). Anticipation from biological motion: The goalkeeper problem. Journal of Experimental Psychology: Human Perception and Performance, 38(4), 848–864. 22309088 10.1037/a0026962

[bibr13-20416695211040237] Dijksterhuis A. Bargh J. A. (2001). The perception–behavior expressway: Automatic effects of social perception on social behavior. In Zanna M. P. (Ed.), Advances in experimental social psychology (Vol. 33, pp. 1–40). Academic Press.

[bibr14-20416695211040237] Dittrich W. H. (1993). Action categories and the perception of biological motion. Perception, 22(1), 15–22. 10.1068/p220015 8474831

[bibr15-20416695211040237] Dittrich W. H. Troscianko T. Lea S. E. G. Morgan D. (1996). Perception of emotion from dynamic point-light displays represented in dance. Perception, 25(6), 727–738. 10.1068/p250727 8888304

[bibr16-20416695211040237] Emery N. J. (2000). The eyes have it: The neuroethology, function and evolution of social gaze. Neuroscience & Biobehavioral Reviews, 24, 581–604. 10940436 10.1016/s0149-7634(00)00025-7

[bibr17-20416695211040237] Farrow D. Abernethy B. (2003). Do expertise and the degree of perception—Action coupling affect natural anticipatory performance? Perception, 32(9), 1127–1139. 10.1068/p3323 14651325

[bibr18-20416695211040237] Fei-Fei L. Iyer A. Koch C. Perona P. (2007). What do we perceive in a glance of a real-world scene? Journal of Vision, 7(1), 1–29. 10.1167/7.1.10 17461678

[bibr19-20416695211040237] Gallup A. C. Chong A. Couzin I. D. (2012). The directional flow of visual information transfer between pedestrians. Biology Letters, 8(4), 520–522. 22456331 10.1098/rsbl.2012.0160 PMC3391476

[bibr20-20416695211040237] Gallup A. C. Hale J. J. Sumpter D. J. T. Garnier S. Kacelnik A. Krebs J. R. Couzin I. D. (2012). Visual attention and the acquisition of information in human crowds. Proceedings of the National Academy of Sciences, 109(19), 7245–7250. 10.1073/pnas.1116141109 PMC3358867 22529369

[bibr21-20416695211040237] Gangestad S. W. Simpson J. A. DiGeronimo K. Biek M. (1992). Differential accuracy in person perception across traits: Examination of a functional hypothesis. Journal of Personality and Social Psychology, 62(4), 688–698. 1583592 10.1037//0022-3514.62.4.688

[bibr22-20416695211040237] Gao Y. Yang F. Frisk M. Hernandez D. Peters C. Castellano G. (2019). Learning socially appropriate robot approaching behavior toward groups using deep reinforcement learning. In *28th IEEE International Conference on Robot and Human Interactive Communication (RO-MAN)*. IEEE.

[bibr23-20416695211040237] Gibson J. J. (1979). The ecological approach to visual perception. Houghton Mifflin.

[bibr24-20416695211040237] Gibson J. J. Pick A. D. (1963). Perception of another person’s looking behavior. The American Journal of Psychology, 76(3), 386–394. 13947729

[bibr25-20416695211040237] Haberman J. Whitney D. (2007). Rapid extraction of mean emotion and gender from sets of faces. Current Biology, 17(17), R751–R753. 17803921 10.1016/j.cub.2007.06.039 PMC3849410

[bibr26-20416695211040237] Haberman J. Whitney D. (2009). Seeing the mean: Ensemble coding for sets of faces. Journal of Experimental Psychology: Human Perception and Performance, 35(3), 718–734. 19485687 10.1037/a0013899 PMC2696629

[bibr27-20416695211040237] Hall J. (2004). Cicero and Quintilian on the oratorical use of hand gestures. The Classical Quarterly, 54(1), 143–160.

[bibr28-20416695211040237] Hayes A. F. Krippendorff K. (2007). Answering the call for a standard reliability measure for coding data. Communication Methods and Measures, 1(1), 77–89.

[bibr29-20416695211040237] Hessels R. S. Benjamins J. S. van Doorn A. J. Koenderink J. J. Holleman G. A. Hooge I. T. C. (2020). Looking behavior and potential human interactions during locomotion. Journal of Vision, 20(10), 1–25. 10.1167/jov.20.10.5 PMC7545070 33007079

[bibr30-20416695211040237] Ickes W. Stinson L. Bissonnette V. Garcia S. (1990). Naturalistic social cognition: Empathic accuracy in mixed-sex dyads. Journal of Personality and Social Psychology, 59(4), 730–742.

[bibr31-20416695211040237] IJsselsteijn W. A. de Ridder H. Freeman J. Avons S. E. (2000). Presence: Concept, determinants, and measurement. In Rogowitz B. E. Pappas T. N. (Eds.), Human vision and electronic imaging V (Vol. 3959, pp. 520–529). International Society for Optics and Photonics.

[bibr32-20416695211040237] JASP Team. (2020). JASP (Version 0.14.1) [Computer software]. Technical report.

[bibr33-20416695211040237] Johansson G. (1973). Visual perception of biological motion and a model for its analysis. Perception & Psychophysics, 14(2), 201–211.

[bibr34-20416695211040237] Kendon A. (1983). The study of gesture: Some remarks on its history. In Deely J. N. Lenhart M. D. (Eds.), Semiotics 1981 (pp. 153–164). Springer: Boston, MA.

[bibr35-20416695211040237] Kenny D. A. Albright L. Malloy T. E. Kashy D. A. (1994). Consensus in interpersonal perception: Acquaintance and the Big Five. Psychological Bulletin, 116(2), 245–258. 7972592 10.1037/0033-2909.116.2.245

[bibr36-20416695211040237] Koenderink J. (2014). The all seeing eye? Perception, 43(1), 1–6. 10.1068/p4301ed 24689127

[bibr37-20416695211040237] Koenderink J. (2019). Vision, an optical user interface. Perception, 48(7), 545–601. 10.1177/0301006619853758 31225771

[bibr38-20416695211040237] Koenderink J. Pinna B. van Doorn A. (2020). Capricious texture of time in awareness and art. Art and Perception, 8(2), 188–236.

[bibr39-20416695211040237] Kret M. E. de Gelder B. (2010). Social context influences recognition of bodily expressions. Experimental Brain Research, 203(1), 169–180. 20401473 10.1007/s00221-010-2220-8 PMC2862946

[bibr40-20416695211040237] Li H. Ji L. Tong K. Ren N. Chen W. Liu C. H. Fu X. (2016). Processing of individual items during ensemble coding of facial expressions. Frontiers in Psychology, 7, 1332. 27656154 10.3389/fpsyg.2016.01332 PMC5013048

[bibr41-20416695211040237] Matsumoto D. (2006). Culture and nonverbal behavior. In Manusov V. Patterson M. L. (Eds.), The SAGE handbook of nonverbal communication (pp. 219–235). Sage Publications: London.

[bibr42-20416695211040237] McArthur L. Z. Baron R. M. (1983). Toward an ecological theory of social perception. Psychological Review, 90(3), 215–238.

[bibr43-20416695211040237] Morris D. (2002). *Peoplewatching: The Desmond Morris guide to body language*. Vintage: London.

[bibr44-20416695211040237] Müller S. Abernethy B. Eid M. McBean R. Rose M. (2010). Expertise and the spatio-temporal characteristics of anticipatory information pick-up from complex movement patterns. Perception, 39(6), 745–760. 10.1068/p6438 20698470

[bibr45-20416695211040237] Müller S. Abernethy B. Farrow D. (2006). How do world-class cricket batsmen anticipate a bowler’s intention? Quarterly Journal of Experimental Psychology, 59(12), 2162–2186. 10.1080/02643290600576595 17095494

[bibr46-20416695211040237] Oliva A. (2005). Gist of the scene. In *Neurobiology of attention* (pp. 251–256). Academic Press.

[bibr47-20416695211040237] Patterson M. L. Iizuka Y. Tubbs M. E. Ansel J. Tsutsumi M. Anson J. (2007). Passing encounters east and west: Comparing Japanese and American pedestrian interactions. Journal of Nonverbal Behavior, 31(3), 155–166.

[bibr48-20416695211040237] Peters C. (2005). Direction of attention perception for conversation initiation in virtual environments. In T. Panayiotopoulos, J. Gratch, R. Aylett, D. Ballin, P. Olivier, & T. Rist (Eds.), *Intelligent virtual agents: 5th international working conference, IVA 2005, Kos, Greece, September 12-14* (Lecture Notes in Computer Science and Artificial Intelligence, number 3661, pp. 215–228). Springer.

[bibr49-20416695211040237] Peters C. Pelachaud C. Bevacqua E. Macini M. Poggi I. (2005). A model of attention and interest using gaze behavior. In T. Panayiotopoulos, J. Gratch, R. Aylett, D. Ballin, P. Olivier, & T. Rist (Eds.), *Intelligent virtual agents: 5th international working conference, IVA 2005, Kos, Greece, September 12-14* (Lecture Notes in Computer Science and Artificial Intelligence, number 3661, pp. 229–240). Springer.

[bibr50-20416695211040237] Pinto D. (2008). Passing greetings and interactional style: A cross-cultural study of American English and Peninsular Spanish. Multilingua, 27(4), 371–388.

[bibr51-20416695211040237] Righart R. De Gelder B. (2008). Recognition of facial expressions is influenced by emotional scene gist. Cognitive, Affective, & Behavioral Neuroscience, 8(3), 264–272. 10.3758/cabn.8.3.264 18814463

[bibr52-20416695211040237] Rousselet G. A. Pernet C. R. Wilcox R. R. (2017). Beyond differences in means: Robust graphical methods to compare two groups in neuroscience. European Journal of Neuroscience, 46(2), 1738–1748. 10.1111/ejn.13610 28544058

[bibr53-20416695211040237] Schönbrodt F. D. Wagenmakers E.-J. (2018). Bayes factor design analysis: Planning for compelling evidence. Psychonomic Bulletin & Review, 25(1), 128–142. 28251595 10.3758/s13423-017-1230-y

[bibr54-20416695211040237] Shklovsky, V. (1917). Art as technique. In Rivkin, J., & Ryan, M., *Literary theory: An anthology (3rd ed)*. John Wiley & Sons Ltd.: Chichester, UK.

[bibr55-20416695211040237] Sweeny T. D. Haroz S. Whitney D. (2013). Perceiving group behavior: Sensitive ensemble coding mechanisms for biological motion of human crowds. Journal of Experimental Psychology: Human Perception and Performance, 39(2), 329–337. 22708744 10.1037/a0028712

[bibr56-20416695211040237] Sweeny T. D. Whitney D. (2014). Perceiving crowd attention: Ensemble perception of a crowd’s gaze. Psychological Science, 25(10), 1903–1913. 25125428 10.1177/0956797614544510 PMC4192023

[bibr57-20416695211040237] Sweeny T. D. Whitney D. (2017). The center of attention: Metamers, sensitivity, and bias in the emergent perception of gaze. Vision Research, 131, 67–74. 28057579 10.1016/j.visres.2016.10.014 PMC5292055

[bibr58-20416695211040237] Thorpe S. Fize D. Marlot C. (1996). Speed of processing in the human visual system. Nature, 381, 520–522. 8632824 10.1038/381520a0

[bibr59-20416695211040237] Todorović D. (2006). Geometrical basis of perception of gaze direction. Vision Research, 46(21), 3549–3562. 16904157 10.1016/j.visres.2006.04.011

[bibr60-20416695211040237] Vanmarcke S. Wagemans J. (2015). Rapid gist perception of meaningful real-life scenes: Exploring individual and gender differences in multiple categorization tasks. i-Perception, 6(1), 19–37. 10.1068/i0682 26034569 PMC4441019

[bibr61-20416695211040237] Võ M. L.-H. (2021). The meaning and structure of scenes. Vision Research, 181, 10–20. https://doi.org/10.1068%2Fi0682 33429218 10.1016/j.visres.2020.11.003

[bibr62-20416695211040237] von Cranach M. Ellgring J. H. (1973). Problems in the recognition of gaze direction. Social Communication and Movement: Studies of Interaction and Expression in Man and Chimpanzee, 4, 419.

[bibr63-20416695211040237] Whitney D. Yamanashi Leib A. (2018). Ensemble perception. Annual Review of Psychology, 69, 105–129. 10.1146/annurev-psych-010416-044232 28892638

[bibr64-20416695211040237] Yamanashi Leib A. Fischer J. Liu Y. Qiu S. Robertson L. Whitney D. (2014). Ensemble crowd perception: A viewpoint-invariant mechanism to represent average crowd identity. Journal of Vision, 14(8), 1–13. 10.1167/14.8.26 PMC4114593 25074904

[bibr65-20416695211040237] Zebrowitz L. A. Collins M. A. (1997). Accurate social perception at zero acquaintance: The affordances of a Gibsonian approach. Personality and Social Psychology Review, 1(3), 204–223. 15659350 10.1207/s15327957pspr0103_2

